# Gorham's Disease of the Mandible

**DOI:** 10.1155/2013/723583

**Published:** 2013-08-26

**Authors:** Rathy Ravindran, Anila Karunakaran

**Affiliations:** ^1^Department of Oral & Maxillofacial Pathology, Azeezia College of Dental Science & Research, Diamond Hills, Meeyannoor, Kollam, Kerala State 691537, India; ^2^Department of Oral & Maxillofacial Pathology, Kannur Dental College, Anjarakandy, Mamba, Kannur, Kerala State 670611, India

## Abstract

Gorham's disease is a rare condition characterized by progressive osteolysis of bone with ultimate total disappearance of bone. The aetiology is undetermined. It may affect any bone of the body, although there is predilection for the pelvis, humerus, axial skeleton, and mandible. Because of the rarity, the disorder goes unrecognized. Various treatment modalities are undertaken with very limited success. We report a case of Gorham's disease of mandible in a 62-year-old man and the literature is reviewed with emphasis on aetiology, diagnosis management.

## 1. Introduction

Gorham's Disease (GD) is a rare disorder characterized by destructive proliferation of vascular channels within the bone and the surrounding soft tissues. The first report of a case of boneless arm was in 1838 and again in 1872 by Jackson. Romer reported first case in jaws of the 13-year-old woman in 1924 [1–4]. The condition was named after two physicians, Lemuel whittington Gorham, MD, and Arthur Purdy Stout, MD.

Various synonyms used in the literature to describe the condition include phantom bone, disappearing or vanishing bone disease, acute spontaneous absorption of bone, hemangiomatosis, lymphangiomatosis, idiopathic osteolysis and Gorham's disease. The condition may affect any part of the skeleton, but most commonly involves the skull, shoulder, and pelvic girdle. About 50 cases of Gorham's disease involving maxillofacial region are reported. Here we report a case of Gorham's disease of the mandible and the literature is reviewed with emphasis on etiology, diagnosis and management.

## 2. Case Report 

**Figure 1:**
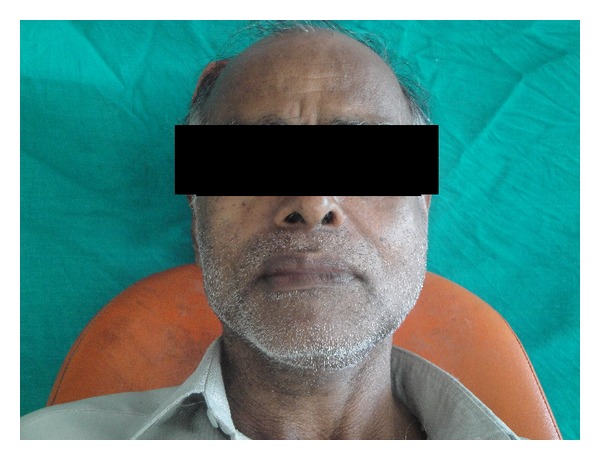
Extraoral examination revealed mild diffuse swelling of left lower face.

**Figure 2:**
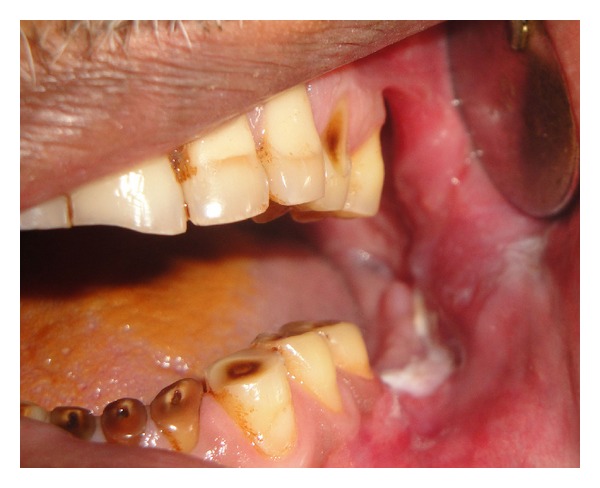
Intraoral examination revealed missing molars.

**Figure 3:**
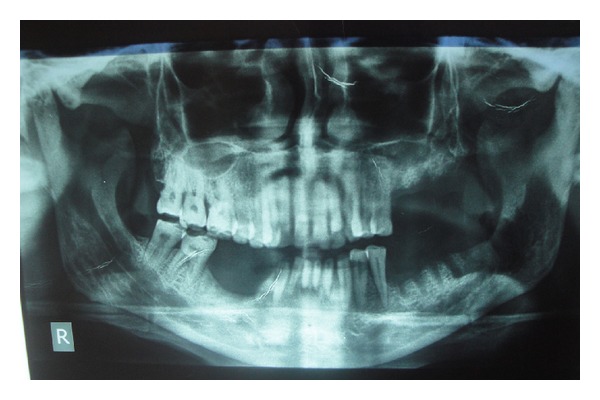
Orthopantomogram (OPG) revealed ill-defined osteolytic region involving body and ascending ramus of mandible.

**Figure 4:**
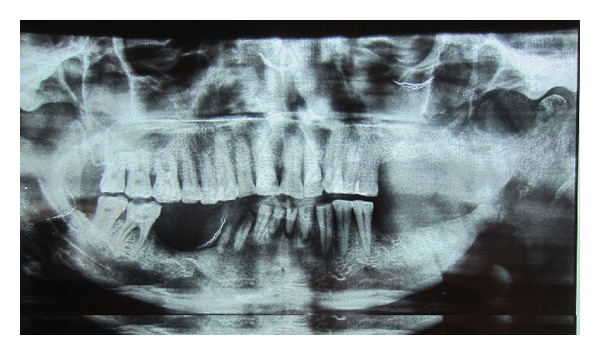
Follow-up OPG taken after 3 months revealed osteolysis extending to involve condyle.

**Figure 5:**
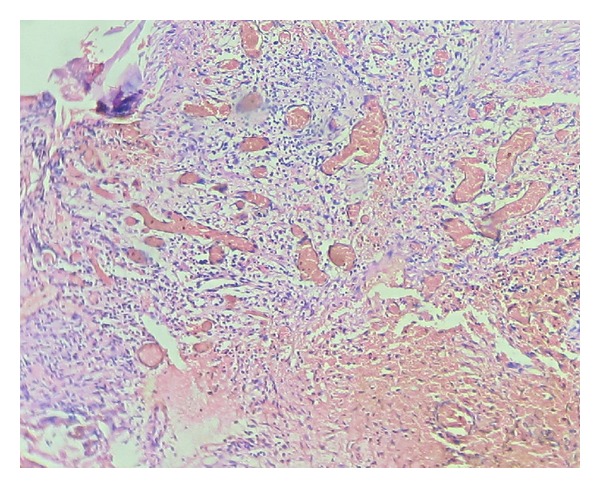
Fibrous connective tissue showing numerous thin walled vascular spaces (H&E 10x).

A 62-year-old male patient reported to the outpatient department with chief complaint of mild discomfort and slight swelling in relation to lower left side of face. The patient gave a history of mobile tooth which was extracted 1 month back. Extra oral examination revealed mild diffuse swelling on lower left side of jaw (Figure 1). The overlying skin was normal. Intraoral examination revealed partially edentulous left mandible with missing molars which was extracted because of mobility (Figure 2). Orthopantomogram revealed an extensive ill-defined osteolytic lesion of body of mandible extending from 37 regions involving angle of mandible and extending up the ramus (Figure 3). Provisional diagnosis of intraosseous malignant neoplasm was made. Differential diagnosis included metastatic tumour, metabolic disease, and osteomyelitis. Hematological investigation of serum calcium, phosphorus, serum alkaline phosphatase and parathyroid hormone levels were found to be within normal range. The laboratory investigation ruled out metabolic disease. The medical and familial histories are noncontributory. Curettage of the affected part was undertaken and the curetted specimen was submitted for histopathological examination. Intraoperative finding suggested complete absence of bone on reflecting the mucosa. The histopathological examination revealed fibrous connective tissue with numerous thin walled vascular spaces and minimal chronic inflammatory infiltrate. No cellular atypia are seen (Figure 5). The histopathological feature was suggestive of angiomatous lesion. The patient was lost to follow up and reported to outpatient department after 3 months with complaint of mild discomfort and an orthopantomogram was advised. A comparison made with previous orthopantomogram revealed osteolytic areas have extended further to involve the condyle (Figure 4). Segmental mandibulectomy was undertaken and the histopathological examination of biopsied sample revealed an angiomatous lesion. Based on patient's history, clinical behavior, histopathological and radiological features diagnosis of Gorham's disease made as per Heffez et al. criteria. The treatment initially undertaken was curettage of the affected part but as the disease progressed, left segmental mandibulectomy was carried out to prevent spread to other areas of bone.

## 3. Discussion

Gorham's disease is extremely rare disorder that may affect any bone but site of predilection is for pelvis, humerus, axial skeleton, mandible. Today more than 150 cases are described in international literature, with more than 50 cases reported involving maxillofacial region [5, 6]. Most cases reported were under 40 years age with mean age of 18 months to 70 years.

The clinical manifestation depend on the site of involvement. Some patients present with abrupt onset of pain and swelling or pathological fracture on the affected site. Gorham's disease of lower jaw will initially affect basal and alveolar bone, which subsequently involves rami and condyle Involvement of temporomandibular joint can be mistaken for Tempromandibular joint dysfunction The clinical course is unpredictable; bone resorption may arrest spontaneously after variable number of years in some cases. Despite the extensive regional loss of bone with resultant deforming atrophy, these patients have only mild disability [7]. The medical and familial histories are noncontributory. The standard laboratory tests are usually within normal limits.

### 3.1. Etiopathogenesis

Today the exact etiology is unknown. The pathological process involves replacement of normal bone by nonneoplastic vascular tissue. Gorham and Stout reported that active hyperemia changes in local PH, and mechanical forces promote bone resorption [4, 5]. Devlin et al. suggested that bone resorption is due to osteoclast activity and interleukin-6 (IL-6) plays a role in increased resorption. Raghuveer and Jayalekshmy suggested that increased osteoclast formation is not due to an increase in circulating precursors but an increase in sensitivity of circulatory precursors to humoral factors [7]. Recent investigation suggested that cells of monocyte-macrophage lineage may play a role in pathogenesis [5].

## 4. Histopathological Features

 The typical histopathological finding is the replacement of bone by connective tissue containing many thin walled blood vessels. It does not represent a hemangioma of bone which is a localised legion. Most authorities are of the opinion that the disease is not due to increased osteoclastic activity, although osteoclasts may be found in the tissues. The absence of osteoclasts in areas of active resorption is often quite striking [8].

 Heffez et al. [9] described the criteria for diagnosis of massive osteolysis as

1. positive biopsy for angiomatous tissue;
2. absence of cellular atypia;
3. minimal or no osteoblastic response and absence of dystrophic calcification;
4. evidence of local progressive osseous resorption;
5. nonexpansile, nonulcerative lesion;
6. absence of visceral involvement;
7. osteolytic radiographic pattern;
8. negative hereditary, metabolic, neoplastic, immunologic, or infectious etiology.

The present case features most of the criteria put forth by Heffez et al. The diagnosis of Gorham's disease is difficult. It must be differentiated from osteomyelitis, hyper parathyroidism, intraosseous malignancies, metastatic disease, eosinophilic granuloma, and osteolysis associated with disease of CNS such as tabes dorsalis, syringomyelia, leprosy, or myelodysplasia.

## 5. Management

Management of Gorham's disease is a challenge for clinicians. Different forms of therapy including medical treatment with oestrogen, calcium, vitamin D, bisphosphonates, and calcitonin, alpha 2B interferon. Surgical treatment options include resection of the lesion, bone grafting, and prosthetic implants. The success of the treatment is very limited. Low dose radiation therapy is controversial and is not accepted as there is chance of radiation induced malignancies [10].

## 6. Conclusion

 Gorham's disease is a rare musculoskeletal disorder. The etiology is undetermined, and currently, there is no effective treatment. It is therefore important for dental surgeons to be aware of its existence as a rare case of osteolysis in maxillofacial skeleton. The diagnosis made based on exclusive of other disease.
